# Ultrasound Evaluation of Subsartorial Spread Following Adductor Canal Block: A Case Series

**DOI:** 10.7759/cureus.60849

**Published:** 2024-05-22

**Authors:** Eric Ly, Kareem Joudi, Vendhan Ramanujam

**Affiliations:** 1 Anesthesiology, Rhode Island Hospital/Warren Alpert Medical School of Brown University, Providence, USA

**Keywords:** regional anesthesia, total knee arthroplasty (tka), ultrasound-guided, subsartorial blocks, adductor canal nerve block

## Abstract

Adductor canal block is a widely used regional anesthesia technique for total knee arthroplasty that helps in reducing post-surgical pain and opioid use. Anatomically, the adductor canal extends from the apex of the femoral triangle proximally to the adductor hiatus distally and is roofed by the sartorius and vasto-adductor fascia. All these serve as a potential path for the spread of the local anesthetic when it is injected inside the adductor canal during the block. Subsartorial space is of unique interest as it lies between the sartorius and vasto-adductor fascia, carrying the subsartorial plexus that can provide additional analgesia to the knee when the adductor canal block injectate spreads into it. While the spread can be variable, ultrasound can be a useful tool to evaluate this spread. This is a case series of patients who underwent total knee arthroplasty under spinal anesthesia and adductor canal blocks. We present the findings of ultrasound evaluation of the injectate spread following adductor canal blocks and evaluation of their analgesia effects.

## Introduction

The utility of ultrasound-guided adductor canal block (ACB) as an integral component of multimodal analgesia for total knee arthroplasty (TKA) is well-recognized. This regional anesthesia technique aims to provide pain relief while preserving motor function and facilitating early postoperative mobilization. The adductor canal is approximately 15 cm long, extending from the apex of the femoral triangle to the adductor hiatus of the adductor magnus. It is bordered anteromedially by sartorius and vasto-adductor (V-A) fascia, laterally by vastus medialis and posteriorly by adductor longus and adductor magnus. It carries the femoral artery, femoral vein, nerve to the vastus medialis, saphenous nerve, and branches of two divisions of obturator nerve. The canal communicates proximally with the femoral triangle, distally with the popliteal fossa, and superficially with subsartorial space. The adductor canal's course and contents provide a conduit for the delivery of local anesthetic agents to block the nerves it carries. However, the effectiveness of ACB is contingent upon the deposition and subsequent spread of these agents. Prior studies have elucidated the pathways of injectate spread into a proximal femoral triangle, distal popliteal fossa, and through fascia [1,2]. While magnetic resonance imaging and dye injection have traditionally been used to evaluate the spread of local anesthetic following a peripheral nerve block, ultrasound can also serve as a feasible tool for this purpose [3]. We report this case series where we evaluated the spread of local anesthetic injectate into the subsartorial space following ACB using ultrasound in patients who arrived for primary TKA. This is unique since such an evaluation using an ultrasound has not been reported.

The findings of this report were previously presented as a meeting abstract at the 49th Annual Regional Anesthesiology & Acute Pain Medicine Meeting on March 21, 2024.

## Case presentation

As the case series is devoid of patient-identifiable information, it is exempt from institutional review board review requirements as per the Lifespan policy. Nevertheless, patient informed consent was obtained for submission. This manuscript adheres to the case reports (CARE) guidelines. This report includes four patients who underwent primary TKA on the same day. None of them had any history of chronic pain or opioid use. For anesthesia, spinal and ACB were planned on all of them. In the preoperative area, their operative side thighs were scanned while in a supine position with the knee semi-flexed and thigh externally rotated using a high frequency (6-13 megahertz) linear probe of an ultrasound (Fujifilm SonoSite, Inc., Bothell, WA) to view the adductor canal borders and their contents both in transverse and sagittal planes. Then, in the same position midway between the inguinal crease and medial condyle of the femur, ACBs were performed using the standard American Society of Anesthesiologists (ASA) monitoring, as needed intravenous midazolam (2-4 mg) and aseptic precautions by the same resident trainee and anesthesiologist. Under ultrasound guidance, using the in-plane needle insertion technique, a 22-gauge x 80-mm echogenic needle (PAJUNK®, SonoPlex® Stim II with Facet Tip, Geisingen, Germany) was inserted towards the lateral position of the femoral artery, and after piercing through the sartorius muscle and V-A fascia, 20 mL of 0.5% bupivacaine was placed [4,5]. After that, ultrasound scanning was again done along the transverse and sagittal planes of the adductor canal, both proximal and distal to the site of local anesthetic injection, to evaluate the spread. The presence of a new anechoic appearance that was absent during primary scanning was identified as the sites of injectate spread. Following the nerve blocks, motor and sensory distribution were checked, after which, spinal anesthesia was performed in the operating room using a 24-gauge x 90-mm pencil point spinal needle (Sprotte®, Geisingen, Germany) and 60 mg of mepivacaine under ASA monitoring by using sterile techniques. The surgery then proceeded with these patients receiving sedation titrated as per their requirement using intravenous propofol infusion.

Case 1

**Figure 1:**
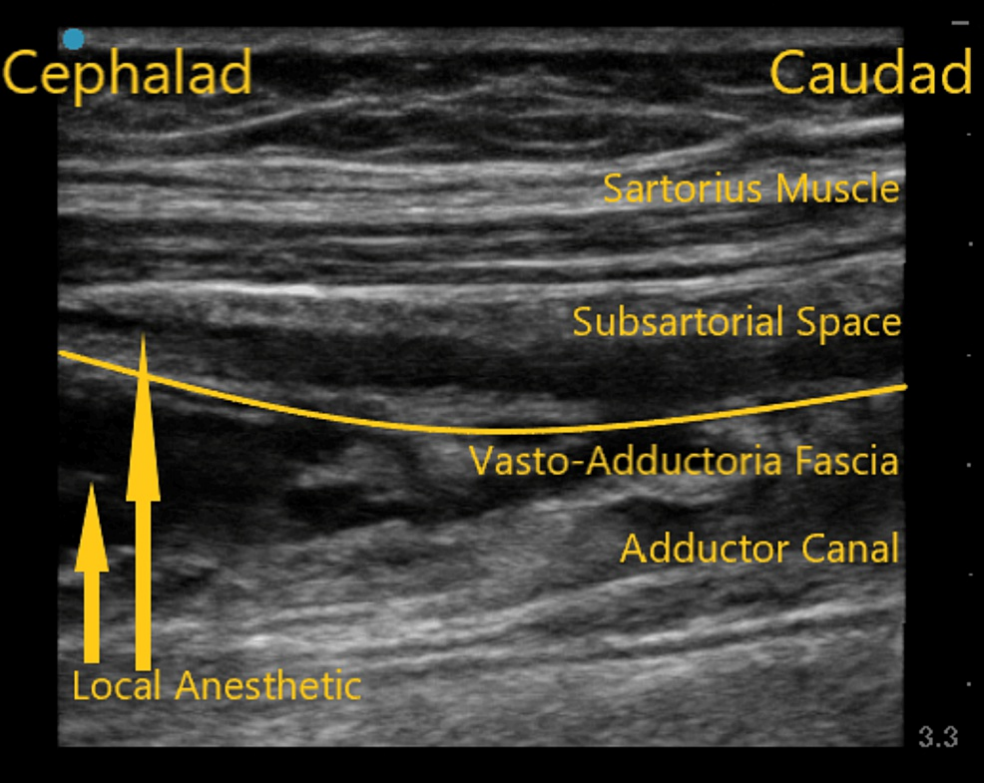
Ultrasound scan image along the sagittal plane of the adductor canal demonstrating subsartorial space and adductor canal spread of local anesthetic.

A 63-year-old male with a body mass index (BMI) of 32.1 kg/m^2^ and ASA physical status of class II presented for his left-sided TKA. His past medical history was significant for obesity and osteoarthritis. His preoperative knee pain score was zero. He was taking as-needed acetaminophen and ibuprofen for his pain control. His physical examination was normal, and laboratory values were within normal limits. Following the ACB, ultrasound scanning demonstrated both the adductor canal and subsartorial spread of the injectate, as shown in Figure 1. Sensory distribution testing revealed nerve blockade distribution along the saphenous and medial cutaneous nerves without any motor weakness in that extremity. His 24-hour postoperative mean pain score was three, and opioid consumption in morphine milli equivalents (MME) was 20 mg.

Case 2

**Figure 2:**
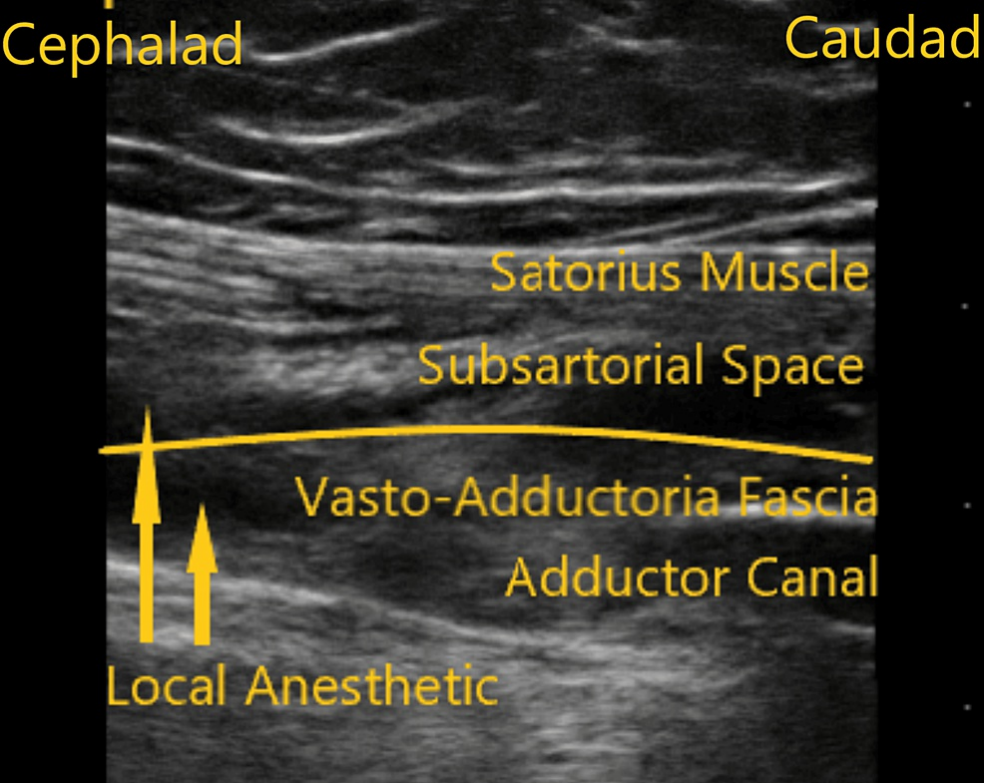
Ultrasound scan image along the sagittal plane of the adductor canal demonstrating subsartorial space and adductor canal spread of local anesthetic.

A 78-year-old female with a BMI of 36.1 kg/m^2^ and ASA physical status of class III presented for her right sided TKA. Her past medical history was significant for coronary artery disease, hypertension, obesity and osteoarthritis. She reported a knee pain score of six. Her medications included metoprolol, hydrochlorothiazide, simvastatin, and, as needed, acetaminophen for pain control. Her physical examination was insignificant, and laboratory values were within normal limits. Figure 2 depicts the ultrasound image of distribution of injectate into the adductor canal and subsartorial space following the ACB. Sensory distribution along the saphenous, medial cutaneous, and obturator nerves were identified without any motor weakness in that extremity. Her 24-hour postoperative mean pain score was 4, and opioid consumption in MME was 7.5 mg.

Case 3

**Figure 3:**
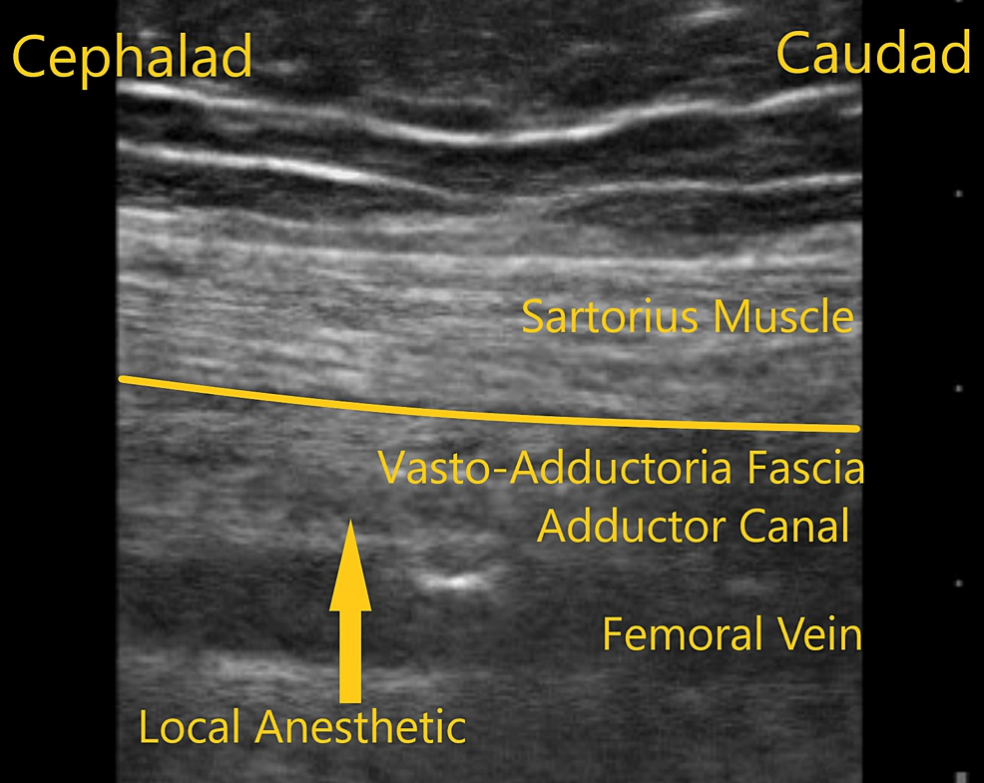
Ultrasound scan image along the sagittal plane of the adductor canal demonstrating only adductor canal spread of local anesthetic.

A 56-year-old female with a BMI of 30.5 kg/m^2^ and ASA physical status of class III presented for her right sided TKA. Her past medical history included coronary artery disease, hypertension, diabetes, obesity, and osteoarthritis. Her knee pain score was 5. Her medications included metoprolol, amlodipine, metformin, and, as needed, acetaminophen for pain control. Physical examination and laboratory values were within normal limits. Figure 3 shows the injectate spread along the adductor canal alone following the block. Sensory distribution was along the saphenous nerve without any lower extremity motor weakness. Here the 24-hour postoperative mean pain score was 5, and opioid consumption in MME was 75 mg.

Case 4

**Figure 4:**
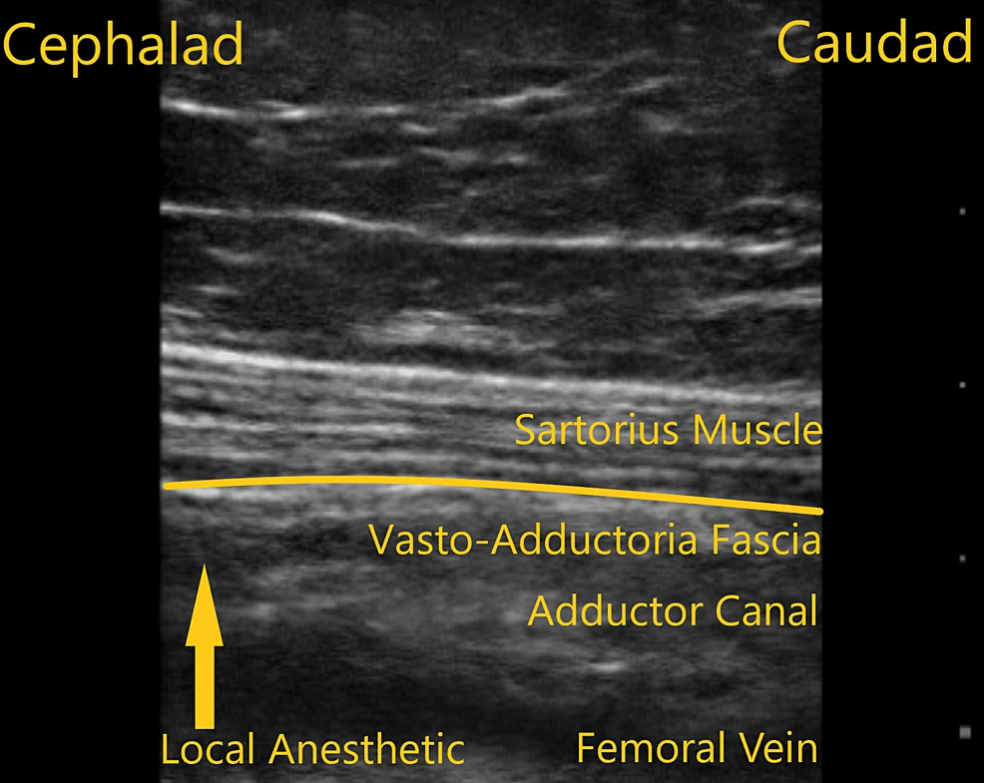
Ultrasound scan image along the sagittal plane of the adductor canal demonstrating only adductor canal spread of local anesthetic.

A 79-year-old female with a BMI of 33.1 kg/m^2^ and ASA physical status of class II presented for her left sided TKA. She had a past medical history of diabetes, obesity, gastroesophageal reflux disease, and osteoarthritis. Her knee pain score was 7. She was on metformin, omeprazole, and, as needed, acetaminophen for pain control. On examination, physical and laboratory values were normal. Figure 4 demonstrates the injectate spread following ACB along the adductor canal alone. Distribution was along the saphenous nerve, and no lower extremity motor weakness was detected. The 24-hour postoperative mean pain score was 6, and opioid consumption in MME was 118.75 mg.

Characteristics of all these patients are included in Table 1.

**Table 1:** Patient characteristics.

Patient	1	2	3	4
Age (years)	63	78	56	79
Sex	Male	Female	Female	Female
Height (cm)	170.2	160	167.6	139.7
Weight (kg)	93	92.5	85.7	64.6
BMI (kg/m^2^)	32.1	36.1	30.5	33.1
ASA	II	III	III	II
Ultrasound Demonstration of Local Anesthetic Spread	Subsartorial and adductor canal	Subsartorial and adductor canal	Adductor canal	Adductor canal
Preoperative Numerical Rating Scale Pain Score	0	6	5	7
Postoperative Mean Numerical Rating Scale Pain Score	3	4	5	6
Postoperative Opioid Medications in Morphine Milli Equivalents (mg)	20	7.5	75	118.75
Sensory Distribution	Saphenous, medial cutaneous	Saphenous, medial cutaneous, obturator	Saphenous	Saphenous
Motor Weakness in Knee and Ankle Flexion or Extension	No	No	No	No

Patients one and two who demonstrated subsartorial local anesthetic spread, along with the adductor canal spread, had lesser postoperative opioid consumption and sensory loss along the distribution of medial cutaneous and obturator nerves in the thigh compared to patients three and four who demonstrated only adductor canal spread. There was no weakness in knee and foot flexion or extension following any of the blocks. They all were discharged in the next 24 hours. In their following-day follow-up, they all reported satisfactory pain control without any complications or side effects either from the peripheral nerve block or from the anesthesia they received.

## Discussion

The roof of the adductor canal is a continuous unbroken fascia, called the fascia V-A, extending from the apex of the femoral triangle to the adductor hiatus that is approximately 26 cm and 7 cm from the base of the patella, respectively. While the fascia’s proximal part is thin quadrangular, the distal part is thick pentagonal. Superficial to this fascia and under the sartorius muscle is the subsartorial space. The V-A fascia is constantly pierced by arterial pedicles from the femoral artery to the sartorius muscle and occasionally by communicating the nerve branches from the saphenous nerve to the medial femoral cutaneous branches above the adductor canal [2]. The spread of the dye injected in the adductor canal into the subsartorial space and vice versa has been demonstrated in the past in cadaver studies [4,6]. Similarly, following an ACB, the spread of the local anesthetic injectate into the subsartorial space from the adductor canal can happen as well, as seen in a couple of patients in this report during evaluation with ultrasound. Since subsartorial space that is a continuation from the femoral triangle is well-defined as a roof in the proximal part of the adductor canal when compared to the canal’s distal portion where the V-A fascia becomes more obvious as a roof [7], injection of local anesthetic in the adductor canal that spreads more distally may not demonstrate a subsartorial space spread pattern when visualized using ultrasound, as seen in the other couple of patients in this report.

The ACB's ability to reduce opioid consumption after TKA surgery is well-known [8]. Subsartorial space has a plexus of nerves that supply the knee joint, which has a complex nerve innervation, including branches from the femoral, obturator, tibial, and common peroneal nerves. The plexus is formed by contributions from the medial cutaneous nerve of the thigh, saphenous nerve, and obturator nerve [5]. During an ACB, besides blockade of the saphenous nerve that runs in the adductor canal, a spread of the local injectate to the subsartorial space can block the nerves of the subsartorial plexus, in addition to providing more analgesia to the knee. This can explain the reduced post-surgery opioid consumption seen in this report in patients who demonstrated the spread of local anesthetic into the subsartorial space in ultrasound imaging compared to those who did not. The sensory-motor distribution achieved following the blocks reminds us that the ACB can provide a reliable sensory blockade of the medial and anterior part of the lower extremity along the saphenous nerve distribution and the block's extension to other nerves is variable due to the anatomical differences that influence the spread rather than the volume of local anesthetic injectate [9].

This report has limitations. This is a case series, and the findings are retrospective, non-comparable to other diagnostic and therapeutic interventions and might be over-interpreted. The subsartorial space spread of a local anesthetic injectate evaluated by using an ultrasound following an ACB and its analgesic effect in TKA cannot be generalized and validated.

## Conclusions

In conclusion, the adductor canal anatomically communicates through interconnections with the superficially lying subsartorial space. This can allow the spread of local anesthetic injectate following an ACB to spread into the subsartorial space through the V-A fascia. Since the subsartorial space carries a plexus of nerves from the femoral, obturator, tibial, and common peroneal nerves that innervate the knee joint, spread to this space following an ACB can contribute to analgesia of the knee joint. This makes ACB a reliable choice for postoperative pain control following knee surgery. Ultrasound is a valuable tool that can be used at the bedside to help detect and learn such spreads following an ACB to determine the effectiveness of the nerve block. This is only a case series, so future human studies investigating the subsartorial spread following an ACB and characteristics of such blocks, especially in regard to sites of injection and volume of injectate, are needed.
